# A Chemical and Enzymatic Approach to Study Site-Specific Sumoylation

**DOI:** 10.1371/journal.pone.0143810

**Published:** 2015-12-03

**Authors:** Claudio P. Albuquerque, Eyan Yeung, Shawn Ma, Ting Fu, Kevin D. Corbett, Huilin Zhou

**Affiliations:** 1 Ludwig Institute for Cancer Research, University of California San Diego, La Jolla, California, United States of America; 2 Department of Cellular and Molecular Medicine, University of California San Diego, La Jolla, California, United States of America; 3 Moores Cancer Center, University of California San Diego, La Jolla, California, United States of America; University of Florida, UNITED STATES

## Abstract

A variety of cellular pathways are regulated by protein modifications with ubiquitin-family proteins. SUMO, the Small Ubiquitin-like MOdifier, is covalently attached to lysine on target proteins via a cascade reaction catalyzed by E1, E2, and E3 enzymes. A major barrier to understanding the diverse regulatory roles of SUMO has been a lack of suitable methods to identify protein sumoylation sites. Here we developed a mass-spectrometry (MS) based approach combining chemical and enzymatic modifications to identify sumoylation sites. We applied this method to analyze the auto-sumoylation of the E1 enzyme *in vitro* and compared it to the GG-remnant method using Smt3-I96R as a substrate. We further examined the effect of *smt3-I96R* mutation *in vivo* and performed a proteome-wide analysis of protein sumoylation sites in *Saccharomyces cerevisiae*. To validate these findings, we confirmed several sumoylation sites of Aos1 and Uba2 *in vivo*. Together, these results demonstrate that our chemical and enzymatic method for identifying protein sumoylation sites provides a useful tool and that a combination of methods allows a detailed analysis of protein sumoylation sites.

## Introduction

SUMO is a member of the ubiquitin-like protein family, and like ubiquitin, is covalently attached to target proteins via an isopeptide bond between the C-terminus of SUMO and the amino group of lysine on the target protein [1,2]. The *SMT3* gene encodes SUMO in *Saccharomyces cerevisiae* and the Smt3 protein is activated by the heterodimeric Aos1-Uba2 complex, the SUMO E1 activating enzyme in *S*. *cerevisiae* [3]. Smt3 is then transferred to Ubc9, the sole SUMO E2 conjugating enzyme [4]. The SUMO E3 ligases Siz1, Siz2 and Mms21 are believed to control sumoylation of specific substrates in cells [5,6]. Like other post-translational modifications, protein sumoylation is reversible and two SUMO-specific isopeptidases, Ulp1 and Ulp2, have been identified in *S*. *cerevisiae* [7,8]. While much is known about the identity and structure of the enzymes that catalyze reversible sumoylation [9], their regulation and substrate selectivity *in vivo* are not well understood.

A key feature of the enzymatic cascade that catalyzes the sequential transfer of ubiquitin family proteins is that modular ubiquitin-fold domains often mediate specific protein-protein interactions to regulate the specificity and efficiency of the transfer reaction [10–12]. Studies of Ubc9 have shown that sumoylation of Ubc9 regulates its activity in *S*. *cerevisiae* and substrate selectivity in humans [13–15]. Other enzymes in the SUMO pathways are also known to be sumoylated, including the Aos1 and Uba2 subunits of the E1 enzyme, Siz1, Siz2, and the Smc5-Smc6-Mms21 complex [6,16,17]. However, it has been unclear whether sumoylation of these enzymes may regulate their activities and/or substrate specificity. Several studies have investigated the role of auto-sumoylation of the E1 enzyme, suggesting that sumoylation of the C terminus of SAE2, the human ortholog of Uba2, is required for its localization to the nuclear periphery, and that sumoylation of the E1’s Cys domain affects the interaction between the E1 and E2 enzymes [18,19]. Despite these studies, further studies are needed to understand the function of E1 and E3 sumoylation.

Hundreds of proteins are known to be sumoylated in *S*. *cerevisiae* [16,17,20–26], and the specific sites at which they are modified have been identified in some cases [27–35]. One approach for identifying sumoylation sites has been the use of a consensus sumoylation motif to predict candidate sumoylation sites that are then evaluated by mutagenesis studies, i.e., the ψKX(D/E) consensus motif, where ψ is a large hydrophobic residue and X is any amino acid [27]. Biochemical studies have shown that this consensus motif interacts with the SUMO E2 enzyme Ubc9 [36–38]. This consensus motif has been successfully used in the analysis of proteins in which relatively few candidate sumoylation sites are present [26,33,39–42]. However, identifying sumoylation sites in large proteins, which can have many candidate sumoylation sites based on this consensus motif alone, requires alternative methods. Moreover, functionally relevant sumoylation sites that do not conform to the above consensus motif have been reported, including K164 of Pol30/PCNA and K153 of Ubc9 [13,28]. A suitable and unbiased method to identify protein sumoylation sites is therefore needed.

Identification of sumoylation sites by MS typically relies on endopeptidase digestion of a sumoylated protein, then detection of a branched peptide with an intact SUMO remnant [27]. For example, trypsin digestion of Smt3 leaves a five amino acid remnant (EQIGG) on the lysine of branched sumoylated peptides, which can be identified by tandem MS analysis [17,21,27]. In the case of mammalian SUMO-1, SUMO-2 and SUMO-3, the use of trypsin would yield a remnant of 19, 19, and 32 amino acids, respectively. In general, as the length of the SUMO remnant increases, the more likely it will fragment during tandem MS analysis to yield fragment ions that are unaccounted for by database search methods, thus compromising the identification. One way to minimize this difficulty is to use alternative endopeptidases to reduce the size of SUMO remnant and chemical modification to improve the sequencing [43,44]. Another way is to create a mutant of SUMO with an arginine preceding the C-terminal Gly-Gly repeat so that a relatively short SUMO remnant, i.e., Gly-Gly, remains attached to sumoylated peptides following trypsin digestion [45], and this strategy has significantly improved the identification of sumoylation sites in both *S*. *cerevisiae* and human cells [45–47]. For example, the use of the *smt3-I96R* mutant in *S*. *cerevisiae* facilitated the identification of 22 sumoylation sites compared to 7 sumoylation sites using WT *SMT3* [45]. However, extensive sequence coverage of sumoylated proteins is often needed to ensure that most, if not all, of the sumoylation sites are identified. The use of endopeptidases with cleavage specificity different from trypsin would improve protein sequence coverage, but would then require a cumbersome re-engineering of SUMO to create a shorter SUMO remnant for the use of each endopeptidase. A general strategy to circumvent these difficulties associated with SUMO remnants is therefore desirable.

We previously reported a proteome-wide approach to identify and quantify sumoylated proteins using quantitative MS [16]. A key feature of our approach was to use the SUMO isopeptidase Ulp1 to facilitate the identification and quantification of lower-abundance sumoylated proteins. However, this approach prevented the identification of sumoylation sites, as Ulp1 treatment generates an unmodified lysine that is indistinguishable from any other unmodified lysine in the protein. As described here, we have adapted this approach to facilitate the identification of the specific lysine on proteins to which SUMO is conjugated. To identify the sites of SUMO conjugation, we first chemically acetylate unmodified lysines on sumoylated proteins. The acetylated sumoylated proteins are then treated with Ulp1 to liberate SUMO, thereby exposing an unmodified lysine at each sumoylation site that can be identified by MS. Using this method we analyzed auto-sumoylation of the E1 enzyme and performed a proteome-wide analysis of protein sumoylation sites in *S*. *cerevisiae*. We further characterized the sumoylation of the E1 enzyme, Aos1 and Uba2, to validate this chemical labeling and Ulp1-cleavage method.

## Results

### A chemical and enzymatic approach for sumoylation site analysis

**Fig 1:**
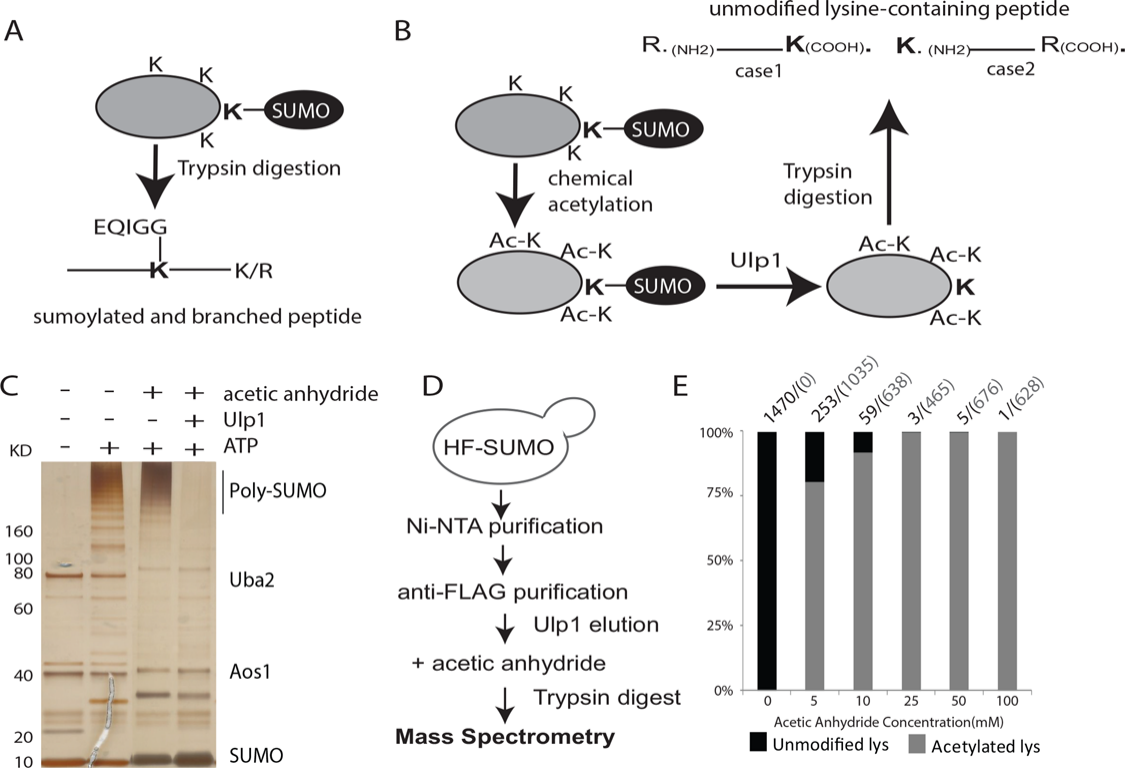
A new chemical and enzymatic approach to analyze protein sumoylation site. (A) Schematic of the conventional method using intact SUMO remnant as a signature for MS analysis. (B) Schematic of our new method (see text for details). (C) *In vitro* sumoylation reaction was performed using purified E1, E2 and Mms21 enzymes to generate poly-SUMO chains. After acetylation, treatment by Ulp1 disassembled poly-SUMO chains. (D) Method to evaluate acetylation efficiency of known SUMO targets purified from yeast cells. Ulp1 was used prior to acetylation reaction. (E) Comparison of acetylated versus unmodified lysine identified for known SUMO targets after treated by increasing concentration of acetic anhydride. The numbers of peptides containing unmodified lysine and acetylated lysine (in parenthesis) are indicated.

Conventional MS-based methods to identify sumoylation sites rely on detection of a SUMO remnant (EQIGG, in *S*. *cerevisiae*) linked to sumoylation sites as a signature (Fig 1A, trypsin is used for illustration purpose). This Smt3 remnant will be fragmented by tandem MS, generating fragment ions that are unaccounted for during database search and thus compromise the sequencing of sumoylated peptides. To overcome this difficulty, we developed a method involving chemical acetylation of all unmodified lysines of SUMO and sumoylated proteins, followed by cleavage with Ulp1 to remove SUMO and generate an unmodified lysine on the targeted protein (Fig 1B). After this two-step treatment, each unmodified lysine specifically marks a sumoylation site. After digestion by trypsin or other endopeptidases, unmodified lysine in linear peptides can be readily identified by MS. Since trypsin specifically recognizes positively charged lysine and arginine, but not uncharged acetylated lysine, we considered two cases of detecting unmodified lysines in trypsin-treated preparations. In Case 1, owing to the trypsin cleavage C-terminal to lysine and arginine residues, an unmodified lysine is detected as the C-terminal amino acid of a peptide. In Case 2, since acetylated lysine is uncharged and hence not recognized by trypsin, any lysine located immediately N-terminal to a detected tryptic peptide is expected to be unmodified, and therefore a candidate sumoylation site.

To address whether Ulp1 could properly cleave SUMO after acetylation, we performed an *in vitro* sumoylation reaction using SUMO E1 (Aos1-Uba2), E2 (Ubc9) and E3 (Mms21) enzymes. Addition of ATP caused an accumulation of poly-SUMO chains, which migrate in a smear-like pattern in an SDS-PAGE gel (Fig 1C, lane 2). *In vitro* sumoylation reaction products were acetylated by treatment with acetic anhydride. Then, one half of the acetylated sample was left untreated (Fig 1C, lane 3), while the other half was treated with Ulp1. We found that treatment of the acetylated sample with Ulp1 resulted in disassembly of poly-SUMO chains (Fig 1C, lane 4). Thus, acetylation of Smt3 did not affect the ability of Ulp1 to disassemble poly-SUMO chains.

To use unmodified lysine as a faithful surrogate of sumoylation site mapping, it is necessary to obtain a complete acetylation prior to Ulp1 treatment. To address this, we expressed 6xHIS-3xFLAG-tagged SUMO (HF-SUMO) at the endogenous *SMT3* locus in yeast cells and purified sumoylated proteins as described previously [16]. The sumoylated proteins were treated with Ulp1 to remove SUMO and eluted from the anti-FLAG resin (Fig 1D). The Ulp1-eluted proteins were then acetylated using increasing concentrations of acetic anhydride, digested by trypsin and analyzed by tandem MS. We then quantified the number of peptides that contained either unmodified lysine or acetylated lysine. As shown in Fig 1E, in the absence of acetic anhydride treatment, virtually all the peptides identified of known SUMO targets contain unmodified lysine (note: SUMO was removed prior to acetic anhydride treatment, Fig 1D). Addition of acetic anhydride drastically reduced the amount of unmodified lysine concurrent with accumulation of acetylated lysine. At concentrations above 25 mM acetic anhydride, very few unmodified lysines were detected, indicating that acetylation had gone to near completion. Additionally, as acetylation approaches completion, the number of total peptides identified decreases (Fig 1E), which could be caused by the inability of trypsin to cleave acetylated lysine within proteins. Because the sizes of peptides are expected to be larger, it is unclear whether this decrease in number of peptides affects protein sequence coverage. We note here that alternative endopeptidases can be used to improve protein sequence coverage and this is made possible by the acetylation and Ulp1-cleavage method. Based on these results all subsequent experiments were conducted using treatment with 50 mM acetic anhydride.

### Identification of sumoylation sites of SUMO E1 enzyme Aos1-Uba2

**Fig 2:**
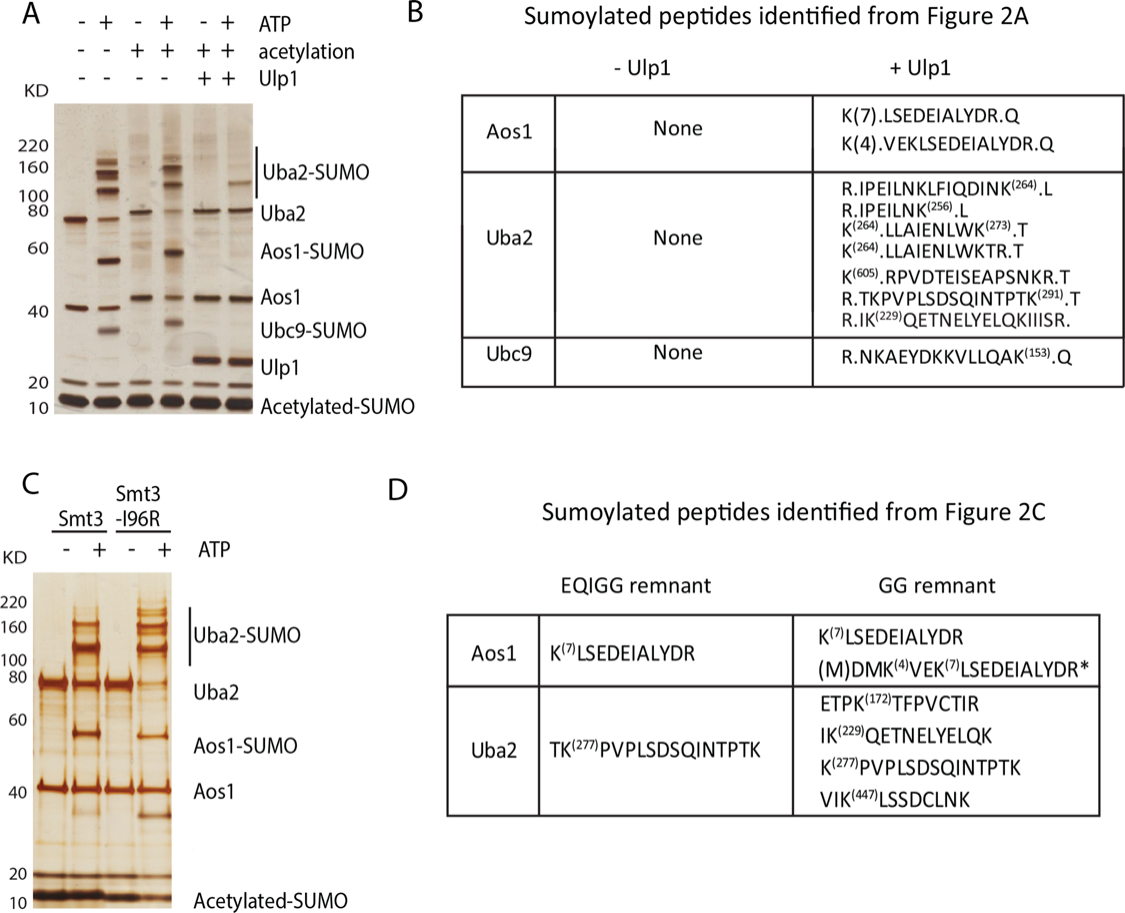
Multiple methods are used to identify sumoylation of E1 and E2 enzymes. (A) *In vitro* sumoylation using E1, E2 and acetylated SUMO, which cannot form poly-SUMO chains, reveals higher molecular weight sumoylated species of Uba2, Aos1 and Ubc9. Ulp1 removal of SUMO allowed the identification of unmodified lysine as sumoylation sites. (B) Peptides that led to the identification of sumoylation sites of E1 and E2 enzymes are listed. Positions of the unmodified lysine (sumoylation site) are shown in parenthesis while all labeled lysine are acetylated. The periods flanking each peptide indicate the site of trypsin cleavage. (C) Comparison between WT Smt3 and Smt3-I96R as substrate for *in vitro* sumoylation by Aos1-Uba2 and Ubc9-K153R. Both WT Smt3 and Smt3-I96R are acetylated to prevent poly-SUMO formation. (D) Sumoylated peptides identified for Aos1 and Uba2 using EQIGG and GG remnants with the position of lysine indicated in parenthesis. * The first methionine was removed from Aos1, which is fused with N-terminal 6xHIS tag for bacterial expression.

To identify sumoylation sites of E1 and E2 enzymes, we performed an *in vitro* sumoylation reaction using purified Aos1-Uba2, Ubc9 and acetylated SUMO (Ac-SUMO). Acetylation of monomeric SUMO with acetic anhydride eliminated the formation of poly-SUMO chains during the *in vitro* reaction. As shown in Fig 2A, multiple sumoylated species of Uba2 were observed, consistent with sumoylation of multiple lysine on Uba2. Additional bands corresponding to sumoylated Aos1 and Ubc9 were also observed. To identify the sumoylation sites of E1 and E2 enzymes, the *in vitro* reaction product was acetylated by the addition of acetic anhydride. Excess glycine was then added to inactivate the remaining acetic anhydride and the sample was split equally into two. In one half of the sample Ulp1 was added to cleave SUMO off and expose an unmodified lysine for each sumoylation site, while the other half of the sample was not treated with Ulp1. Both samples were treated with trypsin and analyzed by MS. As shown in Fig 2B and S2 Table, no unmodified lysine was found for the E1 and E2 enzymes from the sample without Ulp1 treatment, indicating that acetylation is complete. In contrast, several peptides of Aos1 and Uba2 were identified in the Ulp1-treated sample, revealing unmodified lysine as candidate sumoylation sites, including K153 of Ubc9 [13], K4 and K7 of Aos1, K229 and several other lysine of Uba2.

The GG-remnant has been increasingly used for the identification of sumoylation sites, which was first shown by the use of Smt3-I96R in yeast [45]. We sought to compare the use of acetylated Smt3-I96R and WT Smt3 as substrates for *in vitro* sumoylation. For this purpose, we used Ubc9-K153R, which cannot be inhibited by auto-sumoylation [13]. As shown in Fig 2C, both Smt3-I96R and WT Smt3 were readily attached to Uba2 and Aos1. We then digested these reaction products by trypsin and identified sumoylation sites of E1 enzyme using the expected SUMO remnant EQIGG and GG for WT Smt3 and Smt3-I96R, respectively. As summarized in Fig 2D, the use of Smt3-I96R (GG-remnant) allowed for the identification of K4 and K7 of Aos1, K172, K229, K277 and K447 of Uba2, while the use of WT Smt3 (EQIGG-remnant) only identified K7 of Aos1 and K277 of Uba2. Comparison between Fig 2B and 2C shows that the GG-remnant and the Ulp1-cleavage methods are complementary, while the EQIGG-remnant method performs noticeably worse.

### The *smt3-I96R* mutation does not cause adverse *in vivo* defects

**Fig 3:**
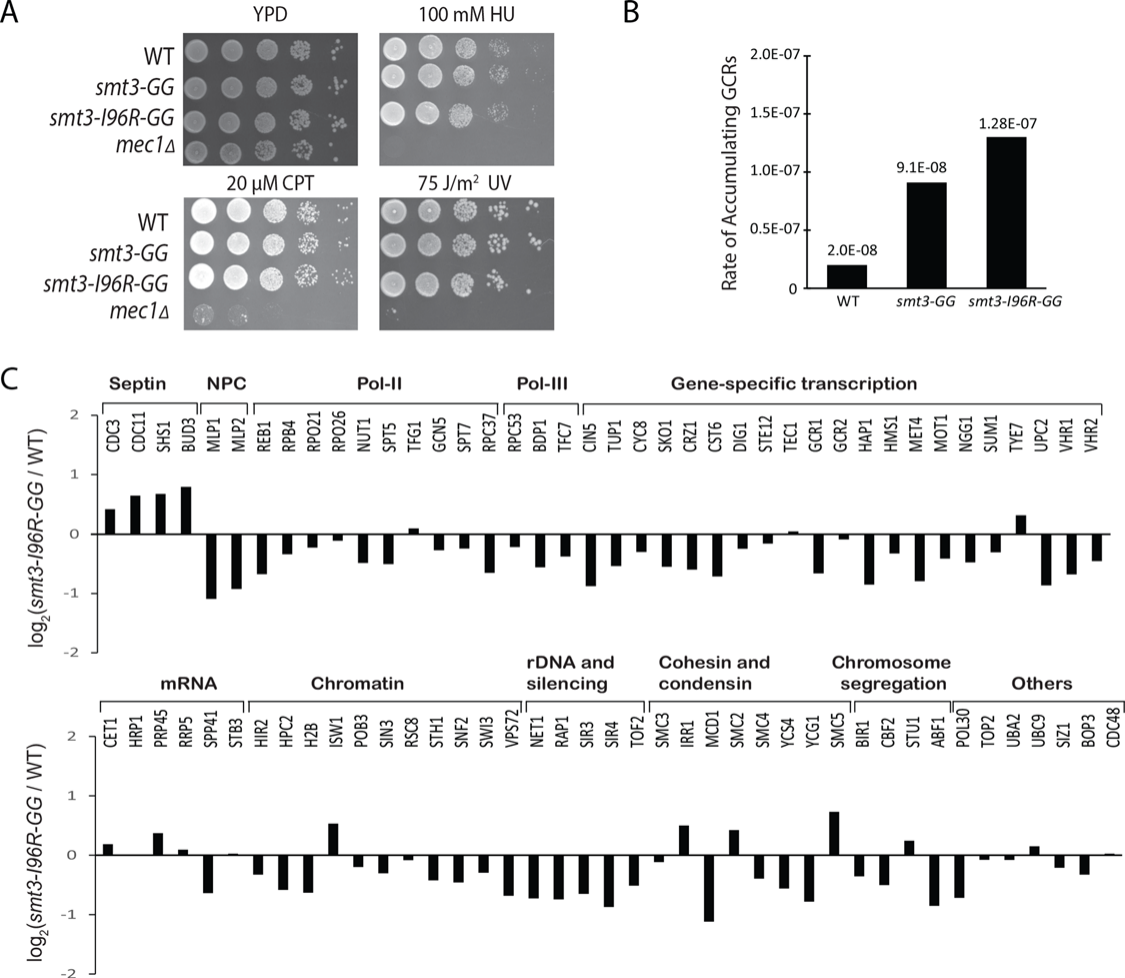
Characterization of the effect of *smt3-I96R* mutation on cell growth, accumulation of GCRs and intracellular sumoylation. (A) Plate assay showed that *smt3-I96R* mutation does not cause any appreciable growth defect in the presence of genotoxic agents. The *mec1Δ* mutant is used as a positive control for its hypersensitivity to genotoxic agents including hydroxyurea (HU), camptothecin (CPT) and UV radiation. (B) Rates of accumulating GCRs in *smt3-I96R* mutant (strain HZY4247) and *smt3GG* mutant (strain HZY3392), both containing a C-terminal stop codon to expose the Gly-Gly repeat and thus mature SUMO in cells. (C) Abundance ratios of the purified sumoylated proteins in WT (HZY2101) and *smt3-I96R* (HZY4248) strains, measured by quantitative MS using the method described previously [16].

Smt3-I96R can substitute for WT Smt3 for *in vitro* sumoylation and Smt3-I96R expressed from a plasmid has been shown to rescue the loss of Smt3, which is essential for cell viability [45]. However, the *in vivo* effect of *smt3-I96R* mutation in yeast has not been fully characterized. To address this, we integrated the *smt3-I96R* mutation in the endogenous locus of *SMT3* and found that it causes no apparent growth defect in response to genotoxic agents (Fig 3A). Considering protein sumoylation has recently been shown to have a major role in preventing gross chromosomal rearrangements (GCRs) [16], we analyzed the effect of *smt3-I96R* mutation and found that the *smt3-I96R* mutation does not cause appreciable change in the rate of GCRs compared to the *smt3-GG* mutation, both yielding the mature form of SUMO in cells, although SUMO maturation mutation causes a modest increase in the rate of accumulating GCRs (Fig 3B). To further examine how Smt3-I96R is attached to intracellular proteins, we performed a quantitative SUMO proteomic analysis to compare wild-type and *smt3-I96R* strain, using the method described previously [16]. As shown in Fig 3C, most known sumoylated proteins show less than two-fold differences in the relative abundance between WT and *smt3-I96R* strain, indicating their sumoylation are not appreciably affected by the *smt3-I96R* mutation. Collectively, these results indicate that the *smt3-I96R* mutation does not cause aberrant cell growth, elevated DNA damage sensitivity and accumulation of GCRs, or appreciable changes to the SUMO proteome in cells. These results support the use of *HF-smt3-I96R* strain for subsequent biochemical studies.

### Proteome-wide identification of sumoylation sites in *S*. *cerevisiae*

**Fig 4:**
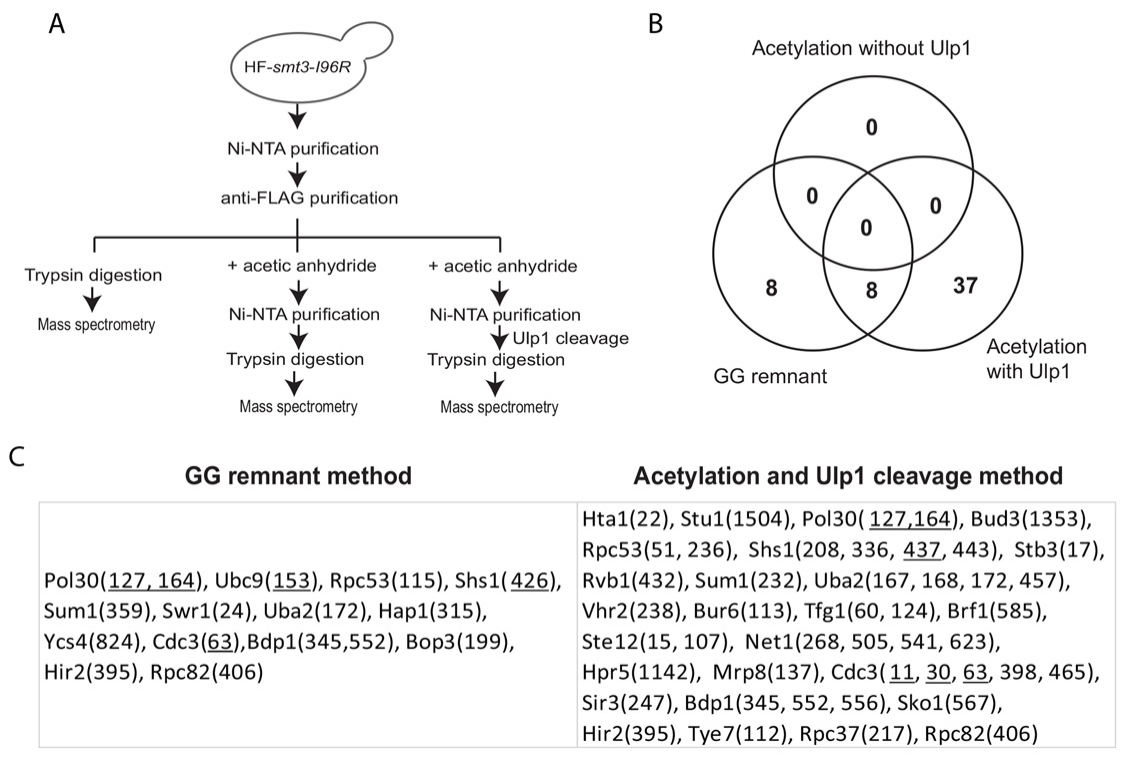
Proteome-wide analysis of protein sumoylation sites using GG-remnant method and the chemical labeling and Ulp1-cleavage method. (A) Sumoylated proteins were purified using Ni-NTA and anti-FLAG affinity resins, eluted by denaturing method and then divided for various treatments as indicated. (B) Venn diagram shows the number of sumoylation sites identified by the GG-remnant method, acetylation without Ulp1 cleavage, and acetylation with Ulp1-cleavage method. (C) Sumoylation sites identified with the position of lysine for each protein shown in parenthesis. Previously known sumoylation sites are underlined.

Next, we sought to identify sumoylation sites of proteins in *S*. *cerevisiae* using the method outlined in Fig 4A. To do so, we purified sumoylated proteins from the *HF-smt3-I96R* strain using first Ni-NTA and then anti-FLAG affinity resins [16]. Sumoylated proteins were eluted from anti-FLAG affinity resins using denaturing buffer containing 6M Urea and the elution was divided equally into three parts (Fig 4A). One-third of the sample was digested with trypsin to generate peptides for MS analysis to identify peptides containing GG-remnants. The remaining two-thirds of the sample was treated with acetic anhydride and then bound to Ni-NTA resins. After washing, this sample was further divided equally into two with one treated with Ulp1 to release proteins from SUMO, while the other sample was not treated with Ulp1. Both samples were digested with trypsin and peptides from all three samples were processed for MS analysis [48]. As summarized in Fig 4B, the GG-remnant method allowed identification of sixteen sumoylation sites, eight of them were also found using the acetylation and Ulp1-cleavage method, which identified many more sumoylation sites. Importantly, no sumoylation site was found of any sumoylated protein in the sample without Ulp1 treatment, indicating that acetylation has gone to completion (see S3 Table for peptide list).

As summarized in Fig 4C, the GG-remnant method identified known sumoylation sites including K127 and K164 of PCNA [28], K153 of Ubc9 [13], K426 of Shs1 and K63 of Cdc3 [27], which are underlined. In addition to identifying PCNA sumoylation sites, the acetylation and Ulp1-cleavage method identified additional known sumoylation sites K11 and K30 of Cdc3 and K437 of Shs1, but not K153 of Ubc9 and K426 of Shs1 [27]. Previous study has shown that mutating these sumoylation sites of Cdc3 and Shs1 did not completely eliminate their sumoylation *in vivo* [27], suggesting that additional sumoylation site(s) exist at a lower stoichiometry. Further study of the new sumoylation sites identified for Cdc3 and Shs1 here could be informative. Sumoylation of PCNA/Pol30 is among the best characterized with only two sumoylation sites K127 and K164 known to date [28]. Importantly, no other lysine was found for Pol30 by either GG-remnant or the acetylation and Ulp1-cleavage method as expected. Using the acetylation and Ulp1-cleavage method, we identified K167, K168 and K172 in the crossover loop of Uba2 (~ 40% sequence coverage), although K229 of Uba2 was not found. The GG-remnant method also allowed the identification of K172 of Uba2 as its sumoylation site. Due to the relatively lower sequence coverage of Aos1 (~ 10%) in this proteomic experiment, we did not identify sumoylation of K4 or K7 of Aos1, likely due to the relative lower abundance of sumoylated Aos1 *in vivo*. We note here that several other known sumoylation sites including K54 of Tec1, K1220 of Top2 and K25 of Gcn5 were not identified here [29,49,50]. Moreover, sumoylation sites of most sumoylated proteins were not identified here, likely due to their relatively lower abundance in unperturbed wild-type cells. Therefore, further studies using specific environmental stimuli or mutations to increase the abundance of sumoylated proteins are necessary for their identification.

### Characterization of Aos1 confirms its sumoylation at K4 and K7

**Fig 5:**
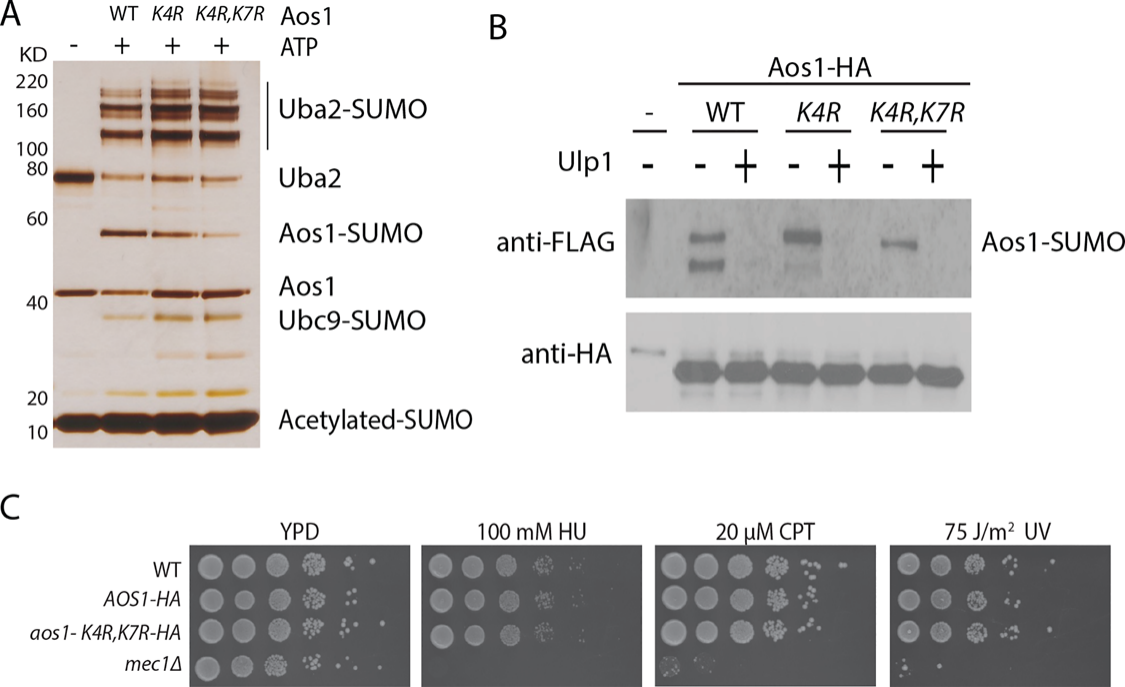
Effect of K4R and K7R mutation on Aos1 sumoylation and function. (A) Effect of K4R and K7R mutations on sumoylation of Aos1 *in vitro*. (B) Effect of Effect of K4R and K7R mutations on Aos1 sumoylation *in vivo*. Epitope-tagged Aos1-HA in the HF-SUMO strain was purified using anti-HA affinity resins and its sumoylated forms was detected using anti-FLAG Western blot. (C) Growth and sensitivity of various sumoylation-defective *aos1* mutants to genotoxic agents. 10-fold dilution of the same amounts of cells were plated and incubated at 30°C for 3 days. The *mec1Δ* mutant is used as a positive control for its hypersensitivity to genotoxic agents including hydroxyurea (HU), camptothecin (CPT) and UV.

Next we chose to confirm the sumoylation sites of the E1 enzyme identified here in order to provide further support for the MS findings and to explore its potential function. To confirm sumoylation of K4 and K7 of Aos1, we purified the Aos1-K4R and Aos1-K4R,K7R proteins from bacteria, which formed a heterodimeric complex with Uba2 during all the purification steps. As shown in Fig 5A, the Aos1 K4R mutation reduced sumoylation of Aos1, and K4R and K7R double mutation further reduced Aos1 sumoylation. Thus, K4 and K7 are its major sumoylation sites *in vitro*. Sumoylation of Uba2 and Ubc9 was not appreciably affected by these mutations, indicating that sumoylation of K4 and K7 of Aos1 is not required for the enzymatic activity of the E1 enzyme.

In our proteome-wide experiment, we did not identify K4 and K7 of Aos1 to be sumoylated due to the low (~ 10%) sequence coverage of Aos1. To evaluate sumoylation of these residues of Aos1 *in vivo*, we generated a strain in which both *HF-SUMO* and *AOS1-3HA* alleles were integrated in their chromosomal loci, respectively [16]. Analysis of Aos1 in whole cell lysate did not reveal any sumoylated species of Aos1, indicating that the stoichiometry of Aos1 sumoylation *in vivo* is very low. Therefore, we chose to enrich Aos1-3HA using anti-HA affinity purification and then analyze the purified sample by immunoblotting. As shown in Fig 5B, two sumoylated bands were found for Aos1 (Fig 5B, lane 2). Ulp1 treatment eliminated both bands, confirming they contain sumoylated Aos1. Interestingly, only the lower but not the upper sumoylated band of Aos1 was reduced by the K4R mutation and eliminated by the K4R and K7R double mutations. The upper sumoylated band of Aos1 is consistent with the presence of multiple sumoylation sites or alternatively one sumoylation site of Aos1 together with an unknown modification *in vivo* (Fig 5A). The latter possibility could exclude its identification by the methods described here. We next examined the phenotype of the *aos1-K4R*, *K7R* double mutant and found no apparent growth defect or elevated sensitivity to genotoxic agents for this mutant (Fig 5C), indicating that auto-sumoylation of K4 and K7 of Aos1 is not essential for its function *in vivo*, although we cannot exclude a possible redundant function of the unknown sumoylation site of Aos1. Taken together, these findings confirmed sumoylation of K4 and K7 of Aos1 *in vitro* and *in vivo*, thus validating our method.

### Multiple sumoylation in the crossover loop of Uba2 occurs *in vivo*

**Fig 6:**
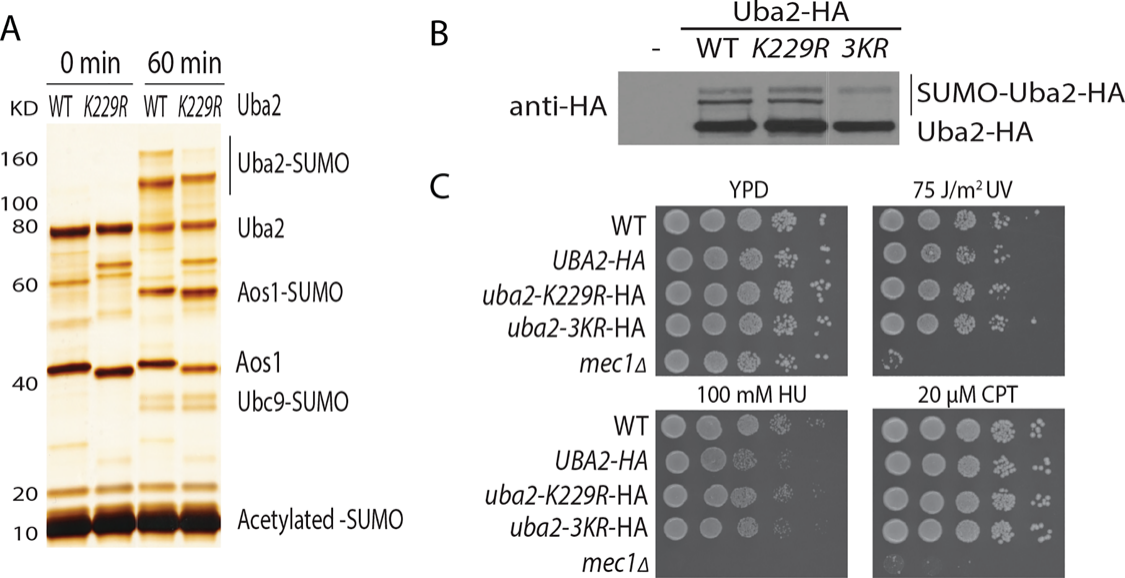
Characterization of Uba2 sumoylation. (A) Effect of *uba2-K229R* mutations on the ability of Aos1-Uba2 in catalyzing sumoylation reaction. (B) Effect of *uba2-K229R* and *uba2-3KR* on Uba2 sumoylation *in vivo*. Whole cell lysates from the indicated yeast strains were subjected to Western blot analysis. (C) Effect of *uba2-K229R* and *uba2-3KR* mutations on cell growth. The *mec1Δ* mutant is used as a positive control for sensitivity to hydroxyurea (HU), camptothecin (CPT) and UV.

Among the sumoylation sites identified for Uba2 following *in vitro* sumoylation reaction (Fig 2), K229 conforms to the known consensus sumoylation motif [27]. The K229-containing peptide of Uba2 was identified via multiple methods (Fig 2), indicating that it is likely a major sumoylation site of Uba2 *in vitro*. To test this, we purified recombinant Uba2-K229R, together with WT Aos1, and tested its sumoylation *in vitro*. As shown in Fig 6A, sumoylation of Uba2 is noticeably reduced, but not eliminated, by the *uba2-K229R* mutation. The presence of additional sumoylated species of Uba2-K229R is consistent with the observation that Uba2 can be sumoylated at multiple lysine *in vitro* (Fig 2).

Instead of analyzing the *in vitro* sumoylation sites of Uba2, which could be artificial, we turned our attention to the sumoylation sites of Uba2 identified from our proteome-wide analysis. As shown in Fig 4 above, several lysines in the crossover loop of Uba2, including K167, K168 and K172, were identified. The crossover loop lies in the active site of Uba2 and is near the catalytic Cys-177 [10]. To examine a possible function for these sumoylation sites, we generated *uba2-3KR* mutant, in which K167, K168 and K172 in the crossover loop of Uba2 were changed into arginine, respectively. We first examined the effect of *uba2-K229R* and *uba2-3KR* mutations on sumoylation of Uba2 in cells. Unlike Aos1 (Fig 5), the abundance of sumoylated Uba2 is sufficiently high and can be readily seen from whole cell lysate without the enrichment of Uba2 (Fig 6B). The *uba2-K229R* mutation has little effect on Uba2 sumoylation *in vivo*, while the *uba2-3KR* mutation specifically eliminates the lower sumoylated specie of Uba2 *in vivo*, confirming the three lysine residues (K167, K168 and K172) in the crossover loop of Uba2 are its major auto-sumoylation sites *in vivo*. We next examined the growth phenotype of these *uba2* mutants and found that the *uba2-3KR* mutant exhibits no obvious growth defects or sensitivity to genotoxic agents (Fig 6C). The lack of growth defect for the *uba2-3KR* mutant suggested that the essential function of Uba2 including its catalytic activity is unlikely affected. Taken together, these results provide further support for the sumoylation sites of Uba2 identified and a validation of our new method.

## Discussion

Protein sumoylation regulates essentially all major nuclear processes in eukaryotic cells and is essential for cell viability. Hundreds of proteins have been found to be sumoylated [17,20–27]; however, relatively little is known about their specific sumoylation sites and the biological function of their sumoylation. Among them, the function of sumoylation of the SUMO E1, E2 and E3 enzymes remains poorly understood except for Ubc9 [13–15]. A major barrier to understanding the functions of sumoylation of the sumoylation enzymes and their substrates has been the lack of suitable methods to identify sumoylation sites. The GG-remnant method was initially reported in yeast and it has later being adopted for mammalian studies [45–47]. Here we report the development of a chemical labeling and Ulp1-cleavage method to identify sumoylation sites, and compare this method with the standard GG-remnant method [45]. We further examined sumoylation of the E1 enzyme Aos1 and Uba2 to validate our new method and evaluate how these methods can be used for biological applications.

Conventional methods to identify sumoylation sites using MS are based on the detection of a branched peptide containing an SUMO remnant, which limits the use of endopeptidases with different sequence specificity. To overcome these problems, we combined the use of chemical labeling and Ulp1 removal of SUMO to generate an unmodified lysine for each sumoylation site, which is readily identified by MS (Fig 1). Acetylation treatment does cause fewer tryptic peptides identified due to the loss of cleavable lysine for trypsin digestion (Fig 1E), although protein sequence coverage may be less affected since peptides are expected to be longer. Nevertheless, removal of SUMO remnant should allow the use of alternative endopeptidases to improve protein sequence coverage if needed. One concern of our new method is that incomplete acetylation would lead to false-positive identification of sumoylation sites. To address this, we evaluated the use of a negative control without Ulp1-treatment, which should yield no unmodified lysine assuming acetylation is complete. Results of this negative control provided further support for the completion of the acetylation reaction and that the sumoylation sites identified using the Ulp1-cleavage method are likely to be correct (Figs 2B and 4B).

Considering that GG-remnant has been increasingly used to identify protein sumoylation sites [45–47], we chose to compare our method with the GG-remnant method (Fig 2). The same sumoylation sites of Aos1, i.e., K4 and K7, were identified using GG-remnant method and our new method, while the EQIGG-remnant method performed noticeably worse. Mutations of both K4 and K7 largely eliminate sumoylation of Aos1, confirming that they are the major sumoylation sites of Aos1 *in vitro*. Compared to Aos1, sumoylation of Uba2 is more complex as it undergoes extensive sumoylation *in vitro* but not *in vivo* (see Figs 2A and 6B), suggesting that some of the *in vitro* sumoylation sites of Uba2 identified could be artificial. While the GG-remnant method allows the identification of K172 in the crossover loop in Uba2 catalytic domain, our new method identified all three lysine in the crossover loop of Uba2, i.e., K167, K168 and K172, as its *in vivo* sumoylation sites (Fig 4B). Further independent validation experiments support this finding, although mutations of these sumoylation sites in the crossover loop of Uba2 do not compromise its essential function in cells (Fig 6).

Several lessons can be gathered from this study concerning the application of sumoylation site mapping methods for a biological study. First, the abundance of sumoylated proteins of interest is critical in order to identify their sumoylation sites by MS regardless of the method used. For example, sumoylation of Uba2 appears to be more abundant than that of Aos1 in unperturbed wild-type cells (see Figs 5 and 6), allowing the identification of *in vivo* sumoylation sites of Uba2 but not Aos1 (Fig 4). Second, a combination of *in vitro* and *in vivo* studies should be pursued to complement each other. For example, identification of K4 and K7 of Aos1 as *in vitro* sumoylation sites prompted us to test and confirm their sumoylation *in vivo*, despite that these sumoylation sites of Aos1 were not identified in our proteome-wide site-mapping experiment (Fig 4). Interestingly, sumoylation of K229 of Uba2 was readily identified from *in vitro* sumoylated Uba2, but not detected in the proteome-wide study. Consistent with this observation, the K229R mutation of Uba2 has little effect on its sumoylation *in vivo*; although we cannot exclude the possibility that sumoylation of K229 may occur at a relatively lower stoichiometry *in vivo*. Despite these progresses, additional sumoylation of Aos1 and Uba2 appear to exist. It is interesting to note that the sumoylation sites found for Uba2 in yeast differ from those found for mammalian SAE2, the ortholog of Uba2 [18,19], raising the possibility that some of these sumoylation sites may have different functions in yeast and human. Third, the GG-remnant method and our new method are complementary and they both should be used for specific applications if needed. It has been shown that anti-GG-remnant antibody could be used to improve the identification of sumoylated peptides [46,47], which we did not use for the study described in Fig 4. Moreover, alternative endopeptidases other than trypsin could be used for the use of our chemical labeling and Ulp1-cleavage method to improve peptide sequence coverage and thus sumoylation site identification if needed. Considering the differences of these methods, we believe they are complementary and should both be used in future biological investigations. Finally, due to the relatively low abundance of most sumoylated proteins in unperturbed wild type cells, their sumoylation sites remain to be identified in our proteomic experiment (Fig 4). One way to overcome this limitation is to apply specific stimuli or mutations to elevate the abundance of specific sumoylated proteins during specific biological investigations regardless of the methods used for sumoylation site identification. In summary, the findings presented here demonstrated that the chemical labeling and Ulp1-cleavage method provides a useful alternative to the standard GG-remnant method for sumoylation site studies.

## Material and Methods

### Yeast genetics method and strains used

Standard yeast genetic and molecular biology methods were used to generate mutations of Aos1 and Uba2. All point mutations of *AOS1* and *UBA2* in yeast were integrated in their respective chromosomal loci and verified by DNA sequencing. A list of yeast strains used in this study is shown in S1 Table.

### *In vitro* sumoylation reaction

For each sumoylation reaction, 0.4 μM Aos1-Uba2 (E1), 2 μM Ubc9 (E2), and 6 μM Smt3 or acetylated Smt3 was used. 0.4 μM Mms21 (E3) was added to the reactions indicated. These proteins were purified using a combination of Ni-affinity, ion exchange and gel filtration columns as previously described [51]. To initiate sumoylation reaction, a final concentration of 1 mM ATP and 2 mM MgCl_2_ was added as indicated. Sumoylation reaction also contains 20mM HEPES pH7.5, 0.15M NaCl and 0.1% NP-40 and it was performed at 30°C for 1 hour unless noted otherwise.

### Acetylation reaction and Ulp1 treatment

To each 100 μl of sumoylation reaction above, 75 mM sodium carbonate and 50 mM acetic anhydride was added sequentially and incubated for 2 hours. To inactivate any residual acetic anhydride, 100 μl of 1M Tris (pH 8.0) was added for 30 min. All of these steps were performed at room temperature. 1 μg of recombinant Ulp1 was then added to cleave SUMO for 2 hours at 30°C.

### Proteome-wide analysis of sumoylation sites using *HF-smt3-I96R* strain

4 liters of *HF-smt3-I96R* cells were grown in YPD to an OD_600nm_ of 1.0 and harvested to purify sumoylated proteins using the method described previously with the following modifications [16]. After the two-step Ni-NTA and anti-FLAG affinity purifications, sumoylated proteins were eluted from anti-FLAG resins by incubating in a buffer containing 6M Urea, 75 mM sodium carbonate and 0.1% NP-40. One third of the eluted sample was diluted 5-fold by PBS buffer and then digested with trypsin for MS analysis. Two-thirds of the eluted sample was incubated with 50 mM acetic anhydride for over 2 hours at room temperature. This sample was then incubated with 100 μl Ni-NTA resins to capture sumoylated proteins. The Ni-NTA resins were then washed with PBS buffer containing 0.2% NP-40 to remove Urea and then divided into two equal portions. One sample was digested with trypsin without Ulp1 treatment, while the other sample was incubated with 1 μg Ulp1 and then digested with trypsin. Following trypsin digestion, all three samples were processed for MS analysis as described previously [48].

### MS data analysis method

The methods for analysis of the tandem mass spectra for peptide identification are the same as those described previously except for the following [16,48]. To search GG-remnant, a variable modification of 114.0429 Dalton on lysine was used. To search EQIGG-remnant, a variable modification of (484.2282 Dalton) on lysine was used. To search for unmodified lysine due to Ulp1-cleavage, we found that among 20 amino acids, tyrosine can also be modified by acetylation to a varying extent, thus variable modifications (42.01057 Dalton) of both tyrosine and lysine by acetylation were used in database search. Additionally, a false discovery rate of 1% was applied to the peptides identified, using a decoy protein database. The search results were then filtered such that only known sumoylated proteins were kept for further analysis, and this is because the same method was used to purify sumoylated proteins from essentially wild-type cells under identical growth condition [16]. Since trypsin was used, we considered both cases in which lysine is either at the C-terminal end of the peptide found or immediately precedes the peptide identified, the latter is based on the known specificity of trypsin to cleave after unmodified lysine and arginine. Finally, we manually inspected the tandem MS spectra for those peptides containing candidate sumoylation sites. For manual inspection, we required that peptides identified are tryptic at both ends with all major ions in the MS/MS spectrum assigned as either Y-ion or B-ion series. The presence of proline should be accompanied by a preferred fragmentation N-terminal to the proline. The assignment of sumoylated lysine is usually unambiguous since it is the only available lysine of the peptide identified. In cases where a tyrosine is present in a peptide, the existence of fragmentation ions that distinguish between lysine and tyrosine was confirmed during manual inspection. Additionally, we have deposited the MS data obtained in this study in a publically accessible database (http://massive.ucsd.edu/ProteoSAFe/result.jsp?task=a0db6d0a63dd4bb6b1684334404e9e5e&view=advanced_view).

### Anti-HA immune-purification method

For Western blot analysis of purified Aos1, 50 ml culture of Aos1-3HA strain was grown in YPD at 30°C to OD_600_ 0.85. Cell pellet was lysed using glass beads method in 0.8 ml of 0.1 M sodium hydroxide, 2% SDS. Whole cell lysate was then neutralized by 0.1 M hydrochloride acid and then 0.1 M Tris pH8.0. The sample was reduced by 10 mM dithiothreitol and alkylated by 40mM iodoacetamide. For immunoprecipitation, 50 μL anti HA-agarose beads (Sigma) was added and incubated for 2 hours at 30°C. Anti-HA beads were washed using TBS-NP40, and the bound proteins were eluted after heated to 100°C in the presence of 1% SDS. To detect Uba2-3xHA sumoylation, which is sufficiently abundant, whole cell lysate was used directly for anti-HA Western blot analysis.

### Phenotype analysis

For plate assay, 10-fold serial dilution of various yeast strains were plated on the indicated plates and grown for up to 3 days at 30°C before pictures are taken. The method used for GCR analysis was described previously [52].

## Supporting Information

S1 Table List of yeast strains used in this study. (PDF)

S2 Table List of peptides identified from MS analysis of *in vitro* sumoylation reaction with or without Ulp1 treatment after acetylation (see Fig 2B). (XLS)

S3 Table List of peptides identified from proteome-wide analysis of *HF-smt3-I96R* strain (see Fig 4). (XLS)
