# Supplementary material for: A Chemical and Enzymatic Approach to Study Site-Specific Sumoylation
Source: PLoS One. 2015 Dec 3;10(12):e0143810. doi: 10.1371/journal.pone.0143810 (PMC4669148; doi:10.1371/journal.pone.0143810)
Supplement: S1 Table — (PDF) [file pone.0143810.s001.pdf]

Supplementary Table 1: List of yeast strains used in this study.

Strain number	Genotype	Study
SCY249	MATa, *ura3-52, leu2∆1, trp1∆63, his3∆200, lys2∆Bgl, hom3-10, ade2∆1, ade8, arg4∆, sml1∆::TRP1*	Chen SH et al. J Biol Chem. 2010
HZY2101	MATa, *6xHIS-3XFLAG-SMT3*, derived from SCY249 with 2 micron removed	Albuquerque CP et al. PLoS Genet. 2013
HZY2131	Isogenic with RDKY6678 containing the GCR assay with 2 micron removed.	Albuquerque CP et al. PLoS Genet. 2013
HZY491	MATa, *AOS1-6xHIS-3xHA::G418,* derived *from* HZY2101	This study
HZY2574	MAT alpha, *mec1Δ::URA, 6xHIS-3XFLAG-SMT3,* derived from HZY2101	This study
HZY3419	MATa, *6xHIS-3XFLAG-SMT3, aos1-K4R-6xHis-3XHA::G418,* derived from HZY2101	This study
HZY3422	MATa, *6xHIS-3XFLAG- SMT3, aos1-K4R, K7R-6xHIS-3XHA::G418,* derived from HZY2101 with 2 micron removed	This study
HZY3499	*MATa, 6xHIS-3XFLAG-SMT3, uba2-K229R-6xHIS-3XHA::HIS,* derived from HZY2101	This study

HZY3504	MATa, *6xHIS-3XFLAG-SMT3, uba2-3KR(K167R, K168R, K172R)-6xHIS-3XHA::HIS,* derived from HZY2101	This study
HZY3392	MAT a, *smt3-GG::G418*, derived from HZY2131. The last three amino acid of Smt3 is removed to expose Gly-Gly repeat.	This study
HZY4247	MAT a, *smt3-I96R-GG::G418*, derived from HZY2131	This study
HZY4248	MAT a, 6xHIS-3xFLAG-*smt3-I96R-GG::G418*, derived from HZY2101	This study
